# Intravitreal Aflibercept injection with Panretinal photocoagulation versus early Vitrectomy for diabetic vitreous hemorrhage: randomized clinical trial

**DOI:** 10.1186/s12886-020-01401-4

**Published:** 2020-04-06

**Authors:** Ahmed Hosni Abd Elhamid, Ahmed Abd El Alim Mohamed, Abeer Mohamed Khattab

**Affiliations:** 1 Ophthalmology Department, Ain Shams University, Cairo, Egypt; 2 Affiliated as vitreoretinal consultant, Hadi hospital, Jabriya, Kuwait; 3 Ophthalmology, Mansoura University, Mansoura, Egypt

**Keywords:** Vitreous hemorrhage, Intravitreal injection, Panretinal photocoagulation, Pars plana vitrectomy

## Abstract

**Background:**

To compare efficacy and safety of intravitreal aflibercept (IVA) injection with panretinal photocoagulation (PRP) versus early vitrectomy for diabetic vitreous hemorrhage (VH).

**Methods:**

Prospective, randomized study that included 34 eyes with diabetic VH. They were divided into two groups, Group Ι (17 eyes) received three successive IVA injections followed by PRP and group ΙΙ (17 eyes) for whom early vitrectomy was done. Follow up was carried out after one, two, three, six and nine months. The primary outcome measure was change in the mean best corrected visual acuity (BCVA) after nine months, secondary outcome measures were mean duration of clearance of VH and rate of recurrent hemorrhage with any additional treatment in both groups. Complications were reported.

**Results:**

There was no statistically significant difference regarding initial demographic criteria between both groups. The mean final log MAR BCVA was statistically better than the initial BCVA in both groups (0.51 ± 0.20, 1.17 ± 0.48 for group I and 0.48 ± 0.18, 1.44 ± 0.44 for group II, *P* < 0.001). There was no statistically significant difference between both groups regarding the mean final Log Mar BCVA (0.51 ± 0.20 for group I, 0.48 ± 0.18 for group II, *p* ≥ 0.05), the mean duration of clearance of VH was 7.8 ± 1.8 weeks, 5 days for group I and II respectively. PRP was completely done for all eyes in group I after three months. The difference in the recurrence rate between group I (29.4%) and group II (11.8%) was statistically significant (*p* < 0.05). Vitrectomy was done for three eyes (17.6%) due to recurrent non-resolving VH in group I. late recurrent VH occurred in two eyes (11.8%) in group II, IVA was given with complete clearance of the hemorrhage. No vision threatening complications were reported in both groups.

**Conclusion:**

Both intravitreal injection of aflibercept followed by PRP and early vitrectomy are effective and safe modalities for treatment of diabetic vitreous hemorrhage. Early vitrectomy leads to faster vision gain with less incidence of recurrence than intravitreal injection.

**Trial registration:**

Randomized clinical trial under the number of NCT04153253 on November 6, 2019 “Retrospectively registered”.

## Background

Diabetic retinopathy is the leading cause of vision loss in working-aged adults worldwide. In proliferative diabetic retinopathy (PDR) stage, hypoxic retinal tissue leads to liberation of angiogenic agents such as vascular endothelial growth factor (VEGF) which plays a major role in the development of retinal and optic disc neovascularization, such neo vessels can bleed at any time and cause vitreous hemorrhage (VH). Although VH occurring as a complication of PDR can cause acute and sometimes severe vision deterioration, there is no current, evidence-based clinical guidance as to what is the best treatment method to provide the best visual outcomes once intervention is needed [1].

For years, pan retinal photocoagulation (PRP) has been the standard of care for high risk PDR and has been shown to reduce severe vision loss by more than 50%^.^ If diabetic VH occurs before detection of the neovascularization, it may be very difficult to do PRP as retinal details are not visible [2].

Anti-VEGF therapy has been shown to cause regression of neovascularization and to potentially prevent further hemorrhage [3]. Recent study from the diabetic retinopathy clinical research network (DRCRN) reported that intravitreal ranibizumab (IVR) is non-inferior to PRP treatment for PDR at the end of two years but the study did not address the role in VH [4]. Another multicenter study compared the outcome of IVR injections versus intravitreal saline injections in the treatment of VH in patients with PDR, and suggested that IVR increased the likelihood of completion of PRP without pars plana vitrectomy (PPV) [5]. Also, there was a beneficial effect of intravitreal bevacizumab (IVB) for managing VH in PDR [6].

PPV is indicated if the hemorrhage does not clear on its own, but surgery does not preclude the possibility of recurrent VH, which has been reported to occur in 20–40% of eyes [7]. Diabetic retinopathy vitrectomy study (DRVS) concluded that there is a benefit of early vitrectomy in eyes of type I diabetics with severe VH and the majority (80%) of patients with type II diabetes with severe VH still require vitrectomy to resolve the VH after 1 year [8]. DRVS was conducted before the development of endolaser photocoagulation which assures complete coagulation of the retina. Further, wide-field visualization, which evolved in the late 1990s, enables more complete endolaser photocoagulation treatment and may allow for better outcomes [9]. Recent studies showed better visual outcome for vitrectomy than results of DRVS, One study showed that the mean visual acuity improved from 20/600 preoperatively to 20/90 postoperatively [10] .

The development of sutureless, micro-incision (23, 25 and 27 gauge) PPV has resulted in less inflammation, increased patient comfort and faster recovery in the post-operative period. Also, with less operative time, the type of anesthesia has been changed from general into local in most of the cases. The improved visual and anatomic outcomes of PPV with current techniques and the reduced post-operative discomfort can encourage retinal surgeons to perform PPV for diabetic VH earlier than three months, which had once been the widely adopted standard for observation [11, 12].

***The aim*** of the study is to compare the efficacy and safety of two different treatment options for diabetic VH, intravitreal aflibecept injection (IVA) with PRP versus early PPV.

## Methods

Prospective randomized study that included patients presenting with diabetic VH in the period from March 2018 to July 2019. The study was done in two eye centers; Hadi hospital in Kuwait and Ain Shams University hospital in Egypt. The study was performed in accordance with the ethical standards of the Declaration of Helsinki and was approved by the Ethical Committee of Hadi hospital.

### Study population

Thirty four eyes of 34 patients with VH as a complication of PDR were selected. *Eligibility criteria* were age above 18 years, any sex, type Ι or ΙΙ diabetes mellitus (DM), recent diabetic VH causing vision impairment and precluding complete PRP, and best corrected visual acuity (BCVA) less than 20/80 (equivalent to log MAR BCVA score of 0.6) and better than 20/1000 (log MAR BCVA of 1.7). *Exclusion criteria* were tractional retinal detachment, previous PRP, history of anti-VEGF therapy within the past two months, neo vascular glaucoma (patients with iris neo vessels and normal intraocular pressure (IOP) were not excluded), subhyaloid clotted hemorrhage, vitreomacular traction and raised fibro vascular proliferations. Patients with diabetic macular edema (DME) at time of presentation (if it can be diagnosed) and those diagnosed at any time of the study whether after IVA injection or after surgery were excluded. Patients with systemic contraindications for anti-VEGF or unstable medical conditions such as uncontrolled hypertension (persistently above 180/110 mmHg) or recent thromboembolic event within the past six months were also excluded from the study.

### Initial assessment

BCVA was measured using Snellen chart which was converted into log MAR, anterior segment slit lamp examination, IOP measurement by Goldmann applanation tonometry, detailed fundus examination with indirect ophthalmoscopy and bio microscopy using 90 D lens if possible*.* B scan ultrasound is required to exclude tractional retinal detachment if details of the retina were not clear by clinical examination.

### Grading of VH

#### Grade I

Mild VH included patients with hazy media, clear or a blurry nerve fiber layer and clear optic disc and retinal vessels.

#### Grade II

Moderate VH included all patients with non-visible nerve fiber layer, blurry optic disc and blurry retinal vessels.

#### Grade III

Severe VH included patients with neither visible optic disc nor retinal vessels.

### Patients’ allocation

Eyes were randomly allocated into either groups by simple coin flip. ***Group Ι*** (17 eyes) was subjected to three monthly doses of IVA injections (EYLEA®; Regeneron Pharmaceutical Inc., Tarrytown, NY, USA and Bayer Healthcare, Berlin, Germany) in a dose of 2 mg/0.05 ml. The first injection was given within a week after presentation. PRP was done after the third injection in case of VH clearance. ***Group ΙΙ*** (17 eyes) was subjected to PPV with endolaser treatment within one week of presentation.

### Interventional techniques

#### Intravitreal injection (IVI)

The technique was done in a sterile operating room. After sterilization and toweling with application of sterile speculum, topical anesthetic (oxybuprocain 0.4%) eye drops was instilled into the eye to be treated. Povidone iodine 5% was instilled into the conjunctiva. Injection of 2 mg /0.05 ml aflibercept was given 4 mm from the limbus in phakic eyes and 3.5 mm in pseudophakic eyes. Vision was checked at the end of the procedure to be at least light perception. Topical antibiotic eye drops were given for five days.

#### Panretinal photocoagulation (PRP)

The treatment was considered complete if 500 μm size burns on the retina were placed no further than 1 to 2 burn widths apart, beginning two disc diameters temporal to the center of macula and one disc diameter nasal to the optic disc, and extending towards the equator for the 12 clock hours. However, 10 clock hours would be sufficient if remaining VH prevents sufficient treatment of the 12 clock hours. The power was adjusted to produce grayish white reaction and pulse duration was set at 100 milliseconds.

#### Pars plana vitrectomy (PPV)

23 gauge PPV was used starting with clearance of anterior vitreous followed by core vitrectomy, peripheral vitrectomy then removal of posterior hyaloid membrane. Vitreous base shaving with 360 scleral depression and endolaser treatment were done for all eyes followed by fluid air exchange, 20% SF6 was used only if there was active significant bleeding during surgery or if there was intraoperative retinal tears. All eyes received a combination of topical steroid and antibiotic eye drops as postoperative treatment.

***Follow up*** was carried out after one day, one week, one month, two months, three months, six months and nine months following the initial procedure whether IVI or vitrectomy. Patients of group Ι were scheduled for subsequent IVIs and PRP during their follow up visits. Patients were advised to report immediately if there was any vision deterioration for the possibility of recurrent bleeding.

**VH clearance** was classified as *complete clearance* with clear posterior pole, optic disc and peripheral retina enough to deliver complete PRP as described above, *partial clearance* was considered if there was unclear posterior pole and optic disc or insufficient peripheral retina to deliver complete PRP and *persistent or non-clearing VH* was considered if the density was the same as time of presentation. *Recurrent VH* was defined as increase in the density of the hemorrhage after complete or partial clearance in addition to worsening visual acuity in comparison to the previous visit.

#### Additional treatment for group Ι

**Fig. 1:**
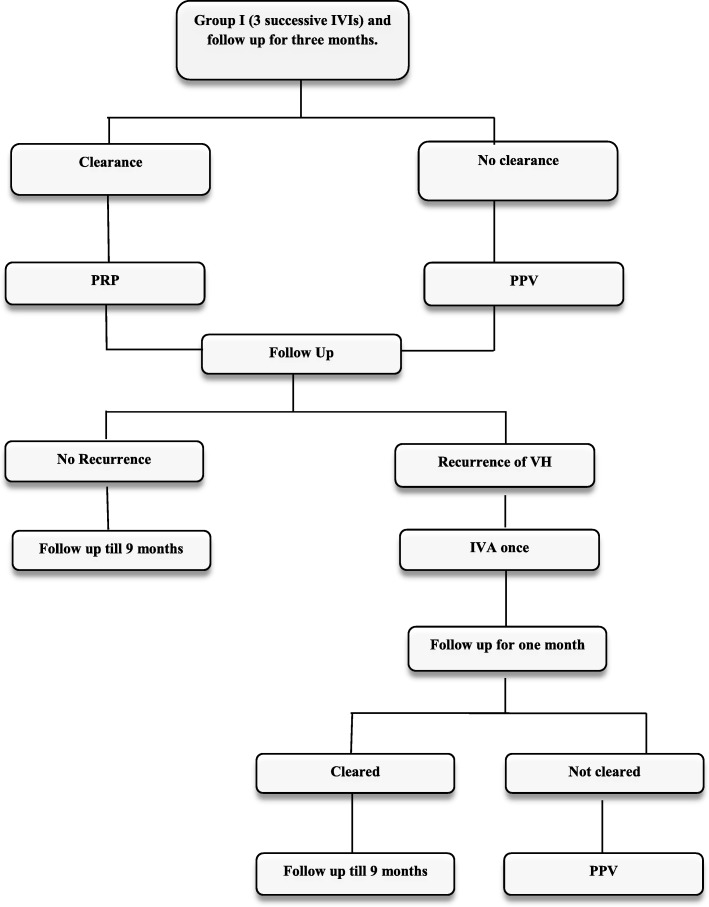
Flow chart for group I

PPV was done for *non-clearing VH or partial clearance* that could not enable complete PRP after three months. If recurrent VH occurred after three IVAs with complete PRP at any time after three months, IVA would be given once more and PPV was indicated if the hemorrhage was not cleared after one month (Fig. 1).

#### Additional treatment for group ΙΙ

**Fig. 2:**
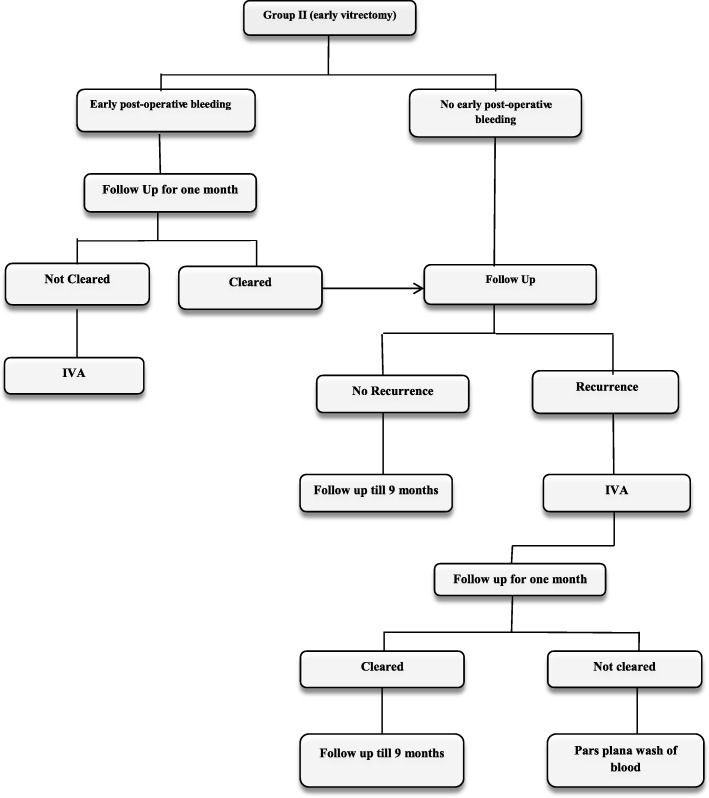
Flow chart for group II

Follow up was done for immediate postoperative VH and IVA was indicated if the hemorrhage was not cleared after one month. If recurrence occurred after the first month, IVA was given with follow up for one month, and if not cleared, pars plana wash of the hemorrhage was indicated (Fig. 2).

The primary outcome measure was change in the mean Log MAR BCVA in both groups after nine months. The secondary outcome measures were duration of clearance of VH, additional required treatment in both groups and report of any complications.

### Data management and statistical analysis

The collected data was revised and introduced to a PC using Statistical package for Social Science (SPSS, Chicago, inc, USA, version 20). Data was presented and suitable analysis was done according to the type of data obtained for each parameter. Parametric numerical data were expressed as mean and standard deviation (SD). *Unpaired T* Test was used to assess the statistical significance of the difference between two study groups’ means (significant difference was considered if *p* ≤ 0.05). Repeated Measure ANOVA test was used to assess the statistical significance of the same variable measure more than two times among the same group.

## Results

There was no statistically significant difference regarding the initial demographic characteristics between both groups (Table 1).

**Table 1:** The initial demographic characteristics of both groups

		Group I (17 eyes)		Group II (17 eyes)		t test	
		Mean/ Nu	SD / %	Mean/Nu	SD / %	*p* value	sig.
**Age (years)**		56.4	8.6	58.8	7.9	0.479	NS
**Gender**	**Male**	8	47.06%	9	52.94%	0.408	NS
	**Female**	9	52.94%	8	47.06%		
**DM type**	**I**	4	23.5%	2	11.8%	0.164	NS
	**II**	13	76.5%	15	88.2%	0.134	
**Duration of DM (years)**		16.4 ±	3.8	15.8 ±	3.9	0.387	NS
**VH Grading**	**I**	2	11.8%	2	11.8%	1	NS
	**II**	9	52.9%	9	52.9%		
	**III**	6	35.3%	6	35.3%		
**Lens status**	**Phakic**	12	70.6%	11	64.7%	0.187	NS
	**Pseudophakic**	5	29.4%	6	35.3%	0.234	NS

### BCVA changes in both groups

**Fig. 3:**
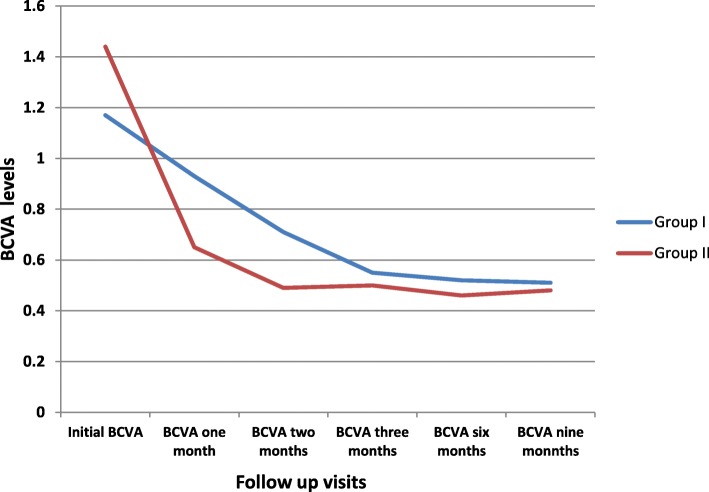
BCVA levels in both groups along study follow up visits. BCVA is measured in log MAR. Repeated measure ANOVA test results: Group I: Initial BCVA versus BCVA at one month was non-significant t (NS), initial versus two, three, six and nine months was significant (S). Group II: Initial versus one, two, three. Six and nine month was significant (S)

The difference was not statistically significant regarding the mean initial log MAR BCVA (1.17 ± 0.48, 1.44 ± 0.44 respectively, *P* = 0.158). At one month, the difference was significantly better in group ΙΙ than group Ι (0.65 ± 0.22, 0.93 ± 0.37 respectively, *p* = 0.039). At two months, the mean log MAR BCVA was also significantly better in group II than group I (0.49 ± 0.19, 0.71 ± 0.2 respectively, *P* = 0.042). The mean BCVA in group I was not statistically different from group II at 3 months (0.55 ± 0.2, 0.50 ± 0.17 respectively, *P* = 0.508). At six months, the difference between both groups was also insignificant (0.52 ± 0.19, 0.46 ± 0.1 respectively, *p* = 0.347). At nine months, the difference was also insignificant between both groups (0.51 ± 0.20, 0.48 ± 0.18 respectively, *p* = 0.412) (Fig. 3)

The mean final BCVA was statistically better than the initial BCVA in group I (0.51 ± 0.20, 1.17 ± 0.48, *P* < 0.001) and the mean final BCVA was statistically better than the initial BCVA in group II (0.48 ± 0.18, 1.44 ± 0.44, *P* < 0.001).

**Fig. 4:**
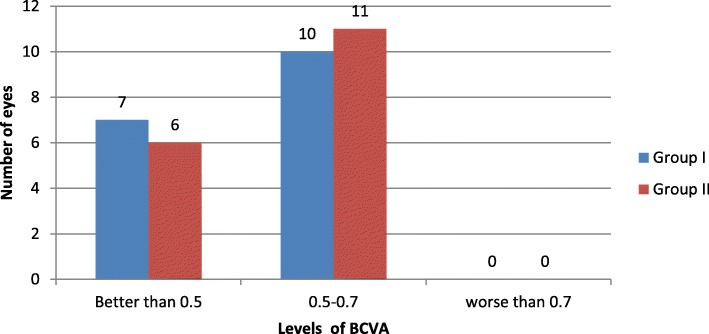
Number of eyes with different final postoperative visual acuity levels in both groups. BCVA levels measured in Log MAR. Treated eyes were categorized according to final log MAR BCVA into three groups (better than 0.5, 0.5–0.7 and worse than 0.7)

There was no significant difference between both groups regarding the number of eyes having final log MAR BCVA better than 0.5, 0. 5-0.7 or worse than 0.7(Fig. 4).

### Clearance of VH

The mean duration of VH clearance in group Ι was 7.8 ± 1.8 weeks while it was 5 days in group ΙΙ (SS, *p* ≤ 0.05).

### Significant complications

Epiretinal membrane (ERM) was found in two eyes (11.8%) in group I and two eyes (11.8%) in group II. Cataract occurred in four out of eleven phakic eyes (36.4%) in group ΙΙ (uneventful phacoemulsification and intraocular lens implantation was done for these eyes in the time between three and six months). Meanwhile, none of the eyes in group I had cataract. Intraoperative retinal tear occurred in two eyes (11.8%) in group II. Significant intraoperative bleeding occurred in one eye (5.9%) in group II. 20% SF6 gas was used in three eyes (17.6%) in group II. Neither intraocular infection nor glaucoma occurred in any eye in both groups. None of the eyes had retinal detachment.

### Additional treatment in both groups for recurrent VH

#### Group I

After three months, clearance of VH with complete PRP was achieved in all eyes (100%). Recurrent VH occurred in **five eyes** (29.4%); the average time for recurrence was 19 weeks. IVA injection was given once after diagnosis of recurrent VH. Two eyes (11.8%) showed cleared VH within a month after the injection and vitrectomy was done for three eyes (17.6%) due to non-clearance of VH after one month from last IVA injection.

#### Group II

Early mild postoperative VH was found in 6 eyes (35.3%) immediately after surgery. All eyes showed complete clearance within the first month, late recurrent VH (after three months) occurred in two eyes (11.8%), IVA injection was given with complete clearance.

The difference in the recurrence rate between group I (29.4%) and group II (11.8%) was statistically significant (*p* < 0.05).

## Discussion

PDR is a common cause of VH. 50% of PDR patients who do not receive timely treatment develop serious vision loss within 5 years [3]. ***Sivaprasad*et al**, [13] studied the effect of repeated aflibercept injection in comparison to standard PRP in cases of PDR without VH and showed that regression of retinal neovascularization at 2 years was seen in 81% of aflibercept-treated eyes and 78% of PRP-treated eyes. 64% of aflibercept-treated eyes had total regression of neovascularization, compared to 34% in PRP eyes. Accordingly, the aflibercept group had greater improvement in retinopathy and showed better BCVA than eyes treated with PRP. ***Gross*et al** [4] showed that IVR was found to be non-inferior to PRP in PDR without VH at the end of two year study.

The present study showed that there was significant improvement of final BCVA in group I which is comparable to ***Alagoz*et al** [14], who used bevacizumab to treat VH, and DRCRN protocol N [5], which showed better visual outcome with IVR in comparison to saline in the short term follow up. ***Huang*et al** [15] found that eyes treated with IVB and those not treated showed similar improvements in BCVA in VH patients who were followed for at least 12 months. In the present study, it was reported that BCVA was improved after IVA in comparison to initial BCVA but it cannot be stated that it would be better than those cases if observed alone as we do not have control group.

Anti-VEGF therapy can be effective for fresh VH; these drugs can cause regression of the neo vessels and prevent recurrent bleeding or new bleeding episode from a new source of neovascularization until spontaneous absorption of the blood occurs, which can permit adequate PRP delivery to the retina with subsequent permanent effect. It reasonable to state that there is correlation between VH clearance and vision improvement with the use of IVA injection. Eyes with DME were excluded initially and throughout the study if it was possible to diagnose them but it is crucial to note that baseline diagnosis of DME can be missed in eyes with VH. Some patients in group I could have associated DME with VH at the beginning of the study which was improved after intravitreal injection and this could be an additive factor for vision improvement in these cases beside VH clearance.

The present study showed complete clearance of VH in 100% of eyes after three injections, which was comparable to ***Spaide and Fisher*** [16], who reported complete clearance of VH after one month following intravitreal anti-VEGF injections in two cases of PDR. ***Alagoz*et al** [14] reported that 86% of his patients showed clearance of VH and completed PRP. Also, ***Elias*et al** [17] reported that 77.7% had completed PRP at the end of follow up after IVR injection. These results are slightly different from the present study which showed that 100% of the eyes had complete PRP after three injections. The difference may be explained by the fact that three successive injections were used even with complete clearance of VH in the present study while ***Alagoz*et al** used anti-VEGF only once and ***Elias*et al** used repeated IVRs for those eyes with incomplete VH clearance. In addition, there may be a difference in the biological effects of different drugs (aflibercept in the present study and bevacizumab in ***Alagoz’s*** study and ranibizumab ***in Elias’s*** study). This may raise a question of whether to inject only once or three successive injections, which is more effective? In case of one injection, it is expected that some patients will clear within a month and others will take longer duration according to many factors as the severity of PDR and density of VH. It is known that after one month there will be no effect of the injected drug so those eyes with non-cleared VH may be subjected to recurrent bleeding exactly as if they were observed for spontaneous clearance. The other option is to inject as needed; meaning, repeated injections are given only if there is incomplete clearance of VH (as done in ***Elias*et al** study) and PRP is done once the hemorrhage is cleared, which may be more convenient for some patients. However, it was preferred in this study to give three successive injections followed by PRP even if VH had been cleared before the second or third injection in order to have as much as possible constant concentration of aflibercept inside the vitreous cavity for all eyes for three months. This would provide a better chance of VH clearance and standardize the protocol of treatment for all eyes with thorough explanation to the patients about the importance of regular follow up and repeating the injections even with clearance of VH.

Recurrent VH occurred in five eyes (29.4%) in group I. DRCR network Protocol N showed that the rate of recurrent VH was 6% for ranibezumab treated eyes and 17% for those eyes who received saline injection however long term recurrence rate cannot be stated in protocol N [18] as the follow up period was only for 16 weeks.

In the past, there has been considerable debating on the timing of performing vitrectomy in patients with diabetic VH. Findings from the DRVS showed that 25% of the early vitrectomy group compared to 15% of the deferral group had a final visual acuity of 20/40 or better after 2 years follow-up. Patients with type I diabetes benefit more from early vitrectomy than patients with type II [8]. Recently, the evolution of small gauge Trans conjunctival vitrectomy is resulting in less operative time, less postoperative inflammation and faster recovery. The development of many surgical instruments with the advancement of equipments and their fluidics encourage most of retinal specialists to do vitrectomy earlier than before. The current study showed that there was significant improvement of BCVA during the follow up visits in comparison to the initial BCVA, which is comparable to other studies. In one series, the mean visual acuity improved from 20/600 preoperatively to 20/90 postoperatively [10]. Another study done by ***Fassbender*et al**, [19] showed that early vitrectomy for VH due to PDR significantly decreases time spent with vision loss.

The present study showed that mean BCVA in group II is better than group I after one and two months (0.93 ± 0.37, 0.65 ± 0.22 after one month 0.71 ± 0.20.49 ± 0.19 after two months) which is expected as surgery usually leads to rapid media clearance in comparison to spontaneous absorption of the blood with the aid of anti-VEGF. There was no difference between the two groups after three, six and nine months.

Although both observation and IVI of anti-VEGF can lead to clearance of vitreous hemorrhage after some variable time, early vitrectomy can be done for patients who seek rapid vision regain and are not comfortable with having poor vision even for one eye for many months as in certain occupations such as drivers, even if VH is being cleared with remaining floater of a blood clot coming intermittently in front of his central vision. Another factor that can make rapid clearance of VH and subsequently rapid vision regain important is the need to regularly monitor other diabetic retinopathy related problems as macular edema or epiretinal membranes.

To our knowledge, this the first published comparative study between anti-VEGF and early vitrectomy for diabetic VH. Although all eyes in both groups had received PRP, the rate of recurrent VH is less in group II than group I. This may be explained by the fact that PPV allows removal of any existing traction on any remaining vascular proliferations and allows doing PRP more anterior than slit lamp delivered PRP, which decreases the chance of recurrent bleeding. Early postoperative bleeding occurred in six eyes (35.3%) in group II, which is comparable to other studies ranging from 20 to 63% [7] and all the eyes showed mild grade one bleeding. The source of this ooze is usually dispersed blood cells from unremoved parts of peripheral vitreous base, especially in phakic eyes. Sclerotomy site may be another source, and mild hypotony that can occur after suturless PPV can lead to ooze from preexisting retinal neovascularization; however, this mild hemorrhage was cleared spontaneously in all eyes within the first month.

The rate of epiretinal membrane formation was similar between both study groups and none of the eyes needed vitrectomy and membrane peel as they were satisfied with their vision. Cataract formation rate was significantly higher in group II than I but it did not affect the final BCVA as uneventful phacoemulsification and intraocular lens implantation was done. 20% SF6 gas was used in three eyes (17.6%). It did not affect the mean BCVA measurement in the first visit after one month as it was completely absorbed by that time.

The current study is limited by the small number of patients in each group and the relatively short period of follow-up.

## Conclusions

Both intravitreal injection of aflibercept and early vitrectomy are effective in treatment of diabetic vitreous hemorrhage with comparable visual outcome. Early vitrectomy can lead to faster vision improvement with less incidence of recurrent hemorrhage, which can be suitable for some patients. Both modalities are equally safe in treatment of diabetic vitreous hemorrhage.

## Data Availability

Data are available with the author upon reasonable request.

## Abbreviations

IVA: Intravitreal Aflibercept
IVR: Intravitreal ranibezumab
IVB: Intravitreal bevacizumab
PRP: Panretinal photocoagulation
VH: Vitreous Hemorrhage
BCVA: Best Corrected Visual Acuity
PDR: Proliferative diabetic retinopathy
PPV: Pars plana vitrrectomy
IOP: Intraocular pressure
DRVS: Diabetic retinopathty vitrectomy study
DM: Diabetes Mellitus
IVI: Intravitreal injection
DME: Diabetic macular edema
VEGF: Vascular endothelial growth factor
